# SARS-CoV-2-Specific T Cell Responses in Immunocompromised Individuals with Cancer, HIV or Solid Organ Transplants

**DOI:** 10.3390/pathogens12020244

**Published:** 2023-02-03

**Authors:** David B. Reeg, Maike Hofmann, Christoph Neumann-Haefelin, Robert Thimme, Hendrik Luxenburger

**Affiliations:** Department of Medicine II (Gastroenterology, Hepatology, Endocrinology and Infectious Diseases), Freiburg University Medical Center, Faculty of Medicine, University of Freiburg, 79106 Freiburg, Germany

**Keywords:** SARS-CoV-2, COVID-19, CD4+ T cells, CD8+ T cells, mRNA vaccination, immunosuppression, cancer, solid organ transplantation, HIV

## Abstract

Adaptive immune responses play an important role in the clinical course of SARS-CoV-2 infection. While evaluations of the virus-specific defense often focus on the humoral response, cellular immunity is crucial for the successful control of infection, with the early development of cytotoxic T cells being linked to efficient viral clearance. Vaccination against SARS-CoV-2 induces both CD4+ and CD8+ T cell responses and permits protection from severe COVID-19, including infection with the currently circulating variants of concern. Nevertheless, in immunocompromised individuals, first data imply significantly impaired SARS-CoV-2-specific immune responses after both natural infection and vaccination. Hence, these high-risk groups require particular consideration, not only in routine clinical practice, but also in the development of future vaccination strategies. In order to assist physicians in the guidance of immunocompromised patients, concerning the management of infection or the benefit of (booster) vaccinations, this review aims to provide a concise overview of the current knowledge about SARS-CoV-2-specific cellular immune responses in the vulnerable cohorts of cancer patients, people living with HIV (PLWH), and solid organ transplant recipients (SOT). Recent findings regarding the virus-specific cellular immunity in these differently immunocompromised populations might influence clinical decision-making in the future.

## 1. Introduction

Since its emergence in December 2019, COVID-19 is a global health burden with more than 660 million confirmed infections causing, in total, 6.7 million deaths worldwide [1]. Of note, the overall numbers are likely to be considerably higher, since it must be assumed that not all cases are reported [2]. The course of SARS-CoV-2 infection is variable and can be asymptomatic or may exhibit symptoms ranging from mild upper respiratory tract infection to multisystem organ failure and death [3]. A rapid diagnosis and differentiation from other common coronaviruses can be achieved with the application of tools such as multiplex PCR [4,5,6]. Additional parameters and techniques, including chest radiography-based evaluations of characteristic alterations in the lung, may further support the assessment of disease severity [3,7,8]. Importantly, in order to improve the treatment of patients with a critical course of disease and the risk of developing an acute respiratory distress syndrome (ARDS), several strategies have been established, among them different (immuno-) therapeutic approaches, personalized clinical monitoring, and individual ventilation management [3,9,10].

In addition to these recent advances, the rapid development of SARS-CoV-2 vaccines, which provide protection via the efficient induction of virus-specific adaptive immune responses, constitutes a milestone in the effort to control the pandemic [11,12,13,14]. In both vaccination and natural infection, the humoral immunity mediated by B cells and antibodies is complemented by cellular responses, which play a crucial role in antiviral defense, as T cells are activated early and associated with effective viral elimination [15,16,17,18,19]. Accordingly, while antibody titers correlate with protection against COVID-19, emerging evidence explicitly connects SARS-CoV-2-specific T cells with a reduced risk of severe disease and death [20,21]. Moreover, and in contrast to the humoral immunity, it appears that this significant role of T cell responses is also valid in the case of exposure to SARS-CoV-2 variants of concern (VOC), since targeted epitopes are largely conserved between the wild-type and VOC, including the Omicron variant [22,23,24,25,26,27].

Importantly, however, most analyses addressing the SARS-CoV-2-specific immunity upon infection or vaccination were performed in healthy and rather young individuals. Consequently, knowledge of virus-specific immune responses in immunocompromised individuals is still limited, despite several reports that point towards a particularly increased risk of severe COVID-19 in this patient group. Considering results from the healthy population, the SARS-CoV-2-specific cellular immunity might be a major factor in determining the degree of protection in this vulnerable cohort. However, first data imply significantly attenuated virus-specific T cell responses following SARS-CoV-2 infection [28,29,30] and vaccination [31,32,33,34,35], in patients suffering from disease-related or iatrogenic immunosuppression. In addition, current guidelines regarding the management of SARS-CoV-2 in these high-risk populations mostly originate from clinical observations, while evidence-based recommendations are limited. Therefore, aiming to propose future research directions and to support clinical decision making, this review discusses recent findings on the T cell immunity induced by SARS-CoV-2 infection and vaccination in immunocompromised individuals, specifically focusing on cancer patients, people living with HIV (PLWH), and solid organ transplant recipients (SOT).

## 2. SARS-CoV-2-Specific T Cells in Healthy Individuals

The coordinated interaction of the different arms of the adaptive immune system, including antibodies, B cells, and T cells, is important for the efficient control of viral infections. Regarding T cells, the antigen is generally presented on MHC class I and class II molecules, thus stimulating antiviral responses by CD8+ or CD4+ T cells [36,37]. This also applies to SARS-CoV-2, where T cells induced by natural infection or vaccination are involved in viral clearance [38,39]. However, the role of cellular immunity, in the context of emerging VOC and after vaccination with second-generation vaccines, has not been fully elucidated and is part of current investigations.

### 2.1. Cellular Immunity during and after SARS-CoV-2 Infection

During and after SARS-CoV-2 infection, virus-specific CD4+ and CD8+ T cell responses develop in addition to antibodies [19]. In healthy subjects, virus-specific T cells directed against various proteins of SARS-CoV-2 (e.g., spike (S), nucleocapsid (N), or membrane (M)) can be widely observed after convalescence and are characterized by a rapid induction, a prolonged contraction, and the formation of a fully functional CD4+ and CD8+ memory T cell pool [15,17,40,41]. During acute SARS-CoV-2 infection, virus-specific CD8+ T cells produce high levels of viral suppression and cytotoxicity effector molecules, such as interferon γ (IFN-γ) and granzyme B, express the activation marker CD107a, and are necessary for the elimination of infected cells [17,41]. On the other hand and in addition to direct viral suppression, particular subsets of virus-specific CD4+ T cells, for instance, T helper 1 cells (T_H_1) or follicular T helper cells (T_FH_), are further involved in the differentiation and maintenance of CD8+ T cells, as well as antibody producing B cells [15,42,43]. Of note, while severe courses of COVID-19 correlate with high levels of SARS-CoV-2-specific antibodies during and after disease, an early generation of strong virus-specific CD4+ and CD8+ T cell responses is associated with milder symptoms [41,44,45].

### 2.2. Cellular Immunity Following SARS-CoV-2 Vaccination

The rapid development of vaccines against SARS-CoV-2 in 2020 constitutes a landmark in the global campaign against the pandemic. Currently applied vaccines are based on different techniques, among them, mRNA, adenoviral vectors, viral proteins, and inactivated viruses. Importantly, all formulations approved by the FDA and EMA are known to provide effective protection against severe COVID-19 via the induction of a strong humoral and cellular immunity targeting the spike protein of SARS-CoV-2 [12,46,47,48,49,50,51,52,53]. In fact, already after two mRNA vaccine doses, healthy individuals generate significant CD4+ and CD8+ T cell responses [13,53,54]. Following the administration of a third and fourth shot, mRNA vaccine-induced CD8+ T cells are temporarily activated and expand. Of note, this boost response lasts for about 30–60 days and the CD8+ T cell memory pool is largely maintained [54]. In line, Swanson et al. describe the development of broad CD4+ and CD8+ T cell responses following two doses of the adenovector-based AZD1222 vaccine, which are characterized by a high degree of polyfunctionality [55]. Finally, as previously mentioned, the epitope repertoire of spike-specific T cells induced by vaccination is mostly preserved in currently circulating VOC, including Omicron [23,56]. Nonetheless, despite these promising results, clinical trials of SARS-CoV-2 vaccination have largely neglected immunocompromised individuals, such as cancer patients, PLWH, or SOT, resulting in an incomplete understanding of the adaptive and, especially, cellular immune response in these particularly endangered cohorts.

## 3. SARS-CoV-2-Specific T Cells in Cancer Patients

### 3.1. Cellular Immunity during and after SARS-CoV-2 Infection

**Figure 1:**
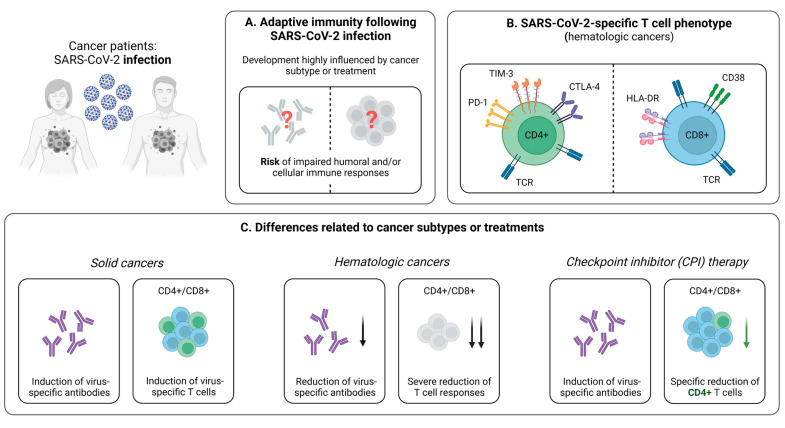
Infection-induced SARS-CoV-2-specific adaptive immune responses in cancer patients. (**A**) Hallmarks of the adaptive immune response following SARS-CoV-2 infection in cancer patients. (**B**) Phenotype of SARS-CoV-2-specific CD4+ and CD8+ T cells in patients suffering from hematologic cancer subtypes. (**C**) Brief summary of current knowledge about the influence of cancer subtypes or specific treatments on the development of SARS-CoV-2-specific adaptive immune responses upon infection. Created with BioRender.com, 2 February 2023.

Cancer patients are at higher risk of developing severe COVID-19, especially if they are elderly and/or share risk factors, such as obesity and the male gender [57,58,59]. Yet, the impact of cancer treatment on the clinical course of infection is largely unclear, with recent studies providing conflicting results [58,60]. Notably, susceptibility towards SARS-CoV-2 appears to depend on the different cancer subtypes [61]. This may be related to varying degrees of impaired adaptive immune responses against SARS-CoV-2, which is particularly striking when comparing patients with solid and hematologic malignancies (Figure 1A).

#### 3.1.1. Reduced Virus-Specific T Cell Responses Following SARS-CoV-2 Infection in Cancer Patients

Cancer patients are characterized by weaker SARS-CoV-2-induced CD4+ and CD8+ T cell responses, compared to healthy individuals [28,29]. This impairment of T cell-mediated immunity is of great clinical relevance, as it correlates with disease progression and may be an influential reason why this group is more likely to suffer from severe COVID-19 [44,45]. Generally, patients with hematological malignancies are at elevated risk for developing upper respiratory tract infections as a result of a substantial immunosuppression [62,63]. This is also the case for COVID-19, where these individuals are known to be particularly endangered [64]. Consequently, it is valuable to consider differences in the cellular immunity as a mediator of disease severity in patients with solid and hematologic malignancies. Indeed, it has been shown that hematologic cancer patients with higher levels of SARS-CoV-2-specific CD8+ T cells during infection have an improved survival, compared to those with a less frequent abundance of this population [29]. Further evaluating T cell responses in patients suffering from solid or hematological malignancies, an interesting study by Fendler et al. described the variations in the immune profiles of cancer patients following natural infection [28]. Thereby, virus-specific CD4+ T cells were detectable in 81% of patients with solid tumors, compared to only 58% in those with hematological malignancies. In line, levels of CD8+ T cells were lower in patients with hematological cancer (42% versus 51% in solid cancer) [28]. Taken together, these results have direct clinical implications for patients with blood cancer and COVID-19, as they represent an exceedingly vulnerable group that might benefit from specific COVID-19 treatments, e.g., monoclonal antibodies and prophylactic SARS-CoV-2 vaccinations.

In addition to its quantity, another relevant aspect of the SARS-CoV-2-specific cellular immune response is the T cell phenotype. Addressing this topic, Bange et al. revealed (1) a similar distribution of CD8+ T cell subsets in subjects with and without cancer, (2) an increased expression of the activation markers CD38 and HLA-DR on CD8+ T cells, and (3) a depletion of CD4+ T cells and B cells in patients with hematologic cancer (Figure 1B) [29]. This is in accordance with reports of Bilich et al., who additionally demonstrated an “exhausted” phenotype of virus-specific CD4+ T cells using flow cytometry-based analyses after intracellular and cell surface marker staining [65]. Hereby, they described higher expression levels of PD-1, CTLA-4, and TIM3 in patients with hematologic malignancies, but not in patients with solid tumors compared to healthy controls (Figure 1B) [65]. In sum, these results highlight the relevance of virus-specific T cells, as they are induced despite an impaired humoral immunity in many cancer patients, which is in line with reports from individuals receiving anti-CD20 treatment [29]. Moreover, considering these cases with an absence of a robust antibody response, the potentially protective role of CD8+ T cells emphasizes the importance of early vaccination against SARS-CoV-2 in cancer patients, with the aim to strengthen the virus-specific cellular immunity [11,12,13,14].

#### 3.1.2. Influence of Cancer Treatment on T Cell Immunity

To date, the influence of different cancer therapies on the SARS-CoV-2-specific immunity remains largely unexplored, with recent studies reporting contradicting results [59,66,67]. Concerning SARS-CoV-2-specific T cells, it has been shown that, among anticancer treatments, only checkpoint inhibitor (CPI) therapy is associated with significantly impaired T cell responses (Figure 1C) [28,68]. Under treatment with CPI, the abundance of detectable SARS-CoV-2-specific CD4+ T cells is reduced, while CD8+ T cell levels remain unaffected [28]. These results are consistent with previous reports in patients with non-small lung cancer, revealing a decrease in CD4+ T cells during treatment with PD-1 inhibitors [68]. Furthermore, they are complemented by studies in respiratory syncytial virus (RSV) infection, which even demonstrate an enhancement of virus-specific CD8+ T cells under CPI therapy [69]. Although the clinical relevance of these findings has not been completely elucidated, a negative effect on the formation of a fully functional adaptive memory is likely, as CD4+ T cell subsets are known to have important effects on various components of the immune system. Prominent examples in this regard are T_H_1 cells promoting CD8+ T cell responses and the formation of a memory pool or T_FH_ cells involved in long-term CD8+ and B cell development [42,70,71,72]. Constituting a precondition to ensure robust immune responses, a functional T cell memory influenced by these factors is decisive, specifically in the case of re-exposure to SARS-CoV-2. Taking all of the discussed findings into account, it is, however, important to note that many current studies addressing the effect of immunotherapy on the SARS-CoV-2-specific immune response included only small numbers of patients. Therefore, further analyses in larger cohorts with longer observation periods are necessary to derive reliable clinical recommendations.

#### 3.1.3. Prolonged SARS-CoV-2 Infection in Cancer Patients

Interestingly, recent reports demonstrate an association of cancer with delayed SARS-CoV-2 clearance (defined as a PCR positivity > 30 days), as well as a link between this prolonged viral shedding and an impaired humoral immunity [73]. These results are in line with observations made in immunosuppressed patients after solid organ transplantation [74]. Comparing, again, solid cancers and hematological malignancies, the latter are associated with a higher viral load and a longer PCR positivity. Furthermore, novel findings suggest a link between prolonged COVID-19 and higher frequencies of fully-functional CD8+ T cells in cancer patients, characterized by a substantial fraction of terminally differentiated effector memory and a lower ratio of central memory T cells [73]. These data indicate that SARS-CoV-2-specific CD8+ T cells are not sufficient for eliminating the virus, but require the interaction with other parts of the adaptive immune system, such as CD4+ T cells, to achieve viral clearance. This is consistent with findings from acute infection in convalescent individuals, implying that a coordinated interplay of virus-specific CD4+ and CD8+ T cells is associated with successful antiviral defense and a milder course of disease [16,41].

### 3.2. Cellular Immunity Following SARS-CoV-2 Vaccination

While numerous studies characterize the vaccine-elicited immunity within the healthy population (see Section 2.2), immune responses in cancer patients are incompletely understood. However, these patients represent a particularly vulnerable cohort, due to concomitant immunosuppression based on diverse causes, including chemotherapy, steroid treatment, or the cancer itself, compromising cellular and humoral immune responses [75]. A detailed assessment of the vaccine-elicited immunity in cancer patients is, therefore, indispensable, as it is required for an evaluation of the vaccine effectiveness and the achievement of best possible protection in this cohort.

#### 3.2.1. Impaired Vaccine-Induced T Cell Immunity in Cancer Patients

**Figure 2:**
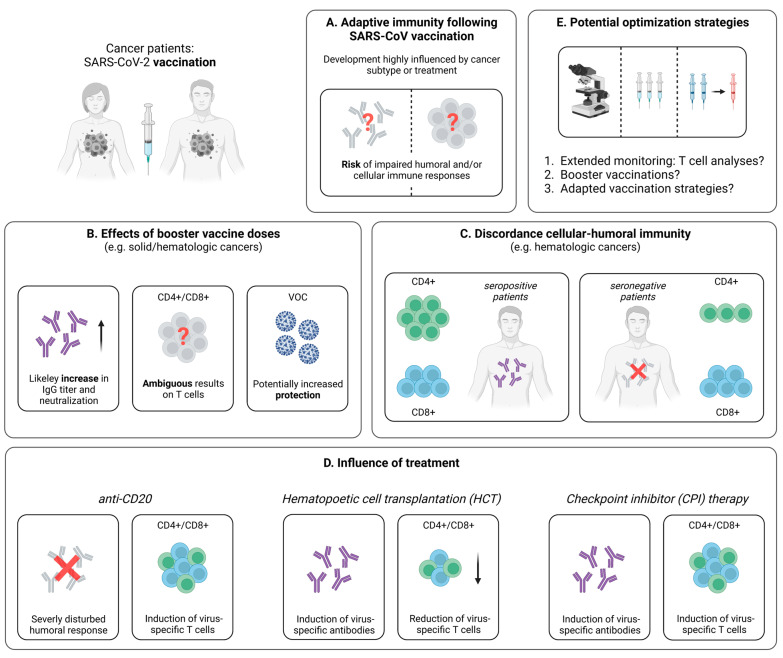
Vaccine-induced SARS-CoV-2-specific adaptive immune responses in cancer patients. (**A**) Hallmarks of the adaptive immune response following SARS-CoV-2 vaccination in cancer patients. (**B**) Collection of first data addressing the effect of booster vaccine doses in patients suffering from different cancer subtypes. (**C**) Comparison of the distribution of cellular immune responses in hematologic cancer patients with or without seroconversion after SARS-CoV-2 vaccination. (**D**) Brief summary of current knowledge about the influence of specific treatments on the development of SARS-CoV-2-specific adaptive immune responses following vaccination. (**E**) Potential tools supporting the assessment or enhancement of vaccine-induced SARS-CoV-2-specific adaptive immune responses in cancer patients. Created with BioRender.com, 2 February 2023.

When compared to healthy individuals, cancer patients elicit lower T cell responses following vaccination with two doses of a mRNA- or vector-based vaccine (Figure 2A). In this regard, Monin et al. detected significantly lower levels of IFN-γ- and IL-2-producing T cells using a FluoroSpot assay in patients with solid and hematological cancer after a single-dose of BNT162b2 [33]. This observation is in line with other studies in cancer patients. For instance, via the application of an ELISPOT assay, Shroff et al. revealed reduced frequencies of IFN-γ-producing T cells after the administration of two doses of a mRNA vaccine, compared to individuals without cancer [76]. In addition to the impaired cytokine secretion following vaccination, T cell responses in cancer patients were also characterized by a more rapid decrease over time [77]. Complementing these findings, Fendler et al. analyzed the adaptive immune response in a large cohort of patients with hematologic and solid cancers who received mRNA- or adenovector-based vaccination [78]. Here, consistent with the results from SARS-CoV-2 infection, a reduced humoral immune response in hematologic cancers, compared to solid cancers, became evident, while similar levels of T cell responses were detected in both cohorts [33,78]. Further emphasizing the importance of virus-specific T cells, some cases of hematologic malignancies are accompanied by a complete lack of humoral responses, despite the administration of two shots of a mRNA- or adenovector-based SARS-CoV-2 vaccine [79,80,81]. Finally, with the intention to provide improved vaccination strategies, McKenzie et al. evaluated the effects of a delayed administration of a second mRNA vaccine dose (>70 days after the first dose) on the adaptive immune response in cancer patients, compared to the standard interval of 21 days [82]. Importantly, no enhancements of the immune response after the delayed second vaccination could be identified. This is contrary to healthy subjects, where the detection of polyfunctional CD4+ and CD8+ T cells via cytokine staining and flow cytometric analyses is comparable in both the standard and the prolonged interval, but antibody titers and neutralizing capacity appear to be superior after a delayed second mRNA vaccine dose [83]. Consequently, these results suggest that cancer patients, as an at-risk group, are likely to benefit from early vaccination with a short interval between the first and second vaccine dose to afford rapid protection against SARS-CoV-2 infection.

#### 3.2.2. SARS-CoV-2-Specific T Cell Immunity upon Booster Vaccination

At present, most studies dealing with cancer patients focus on the adaptive immune response following one or two doses of a SARS-CoV-2 vaccine. However, as these patients are threatened by a lower immunogenicity of this application scheme, considerable benefits might be achieved with the help of booster vaccinations against SARS-CoV-2. Hence, a detailed understanding of the immune response after three and more vaccine doses is necessary to define the optimal vaccination strategies for this vulnerable population. Indeed, the effect of booster vaccinations on the humoral immune response has been studied in more detail (reviewed in [84]); however, the impact on cellular immunity remains somewhat elusive. For patients with solid cancers, Shroff et al. could demonstrate via an ELISPOT approach that a third shot of BNT162b2 results in a boost of the antibody response, but does not improve the frequency of virus-specific T cells (Figure 2B) [76]. In contrast, other reports point towards enhanced T cell responses detected by an IFN-γ ELISPOT assay after a third mRNA- or adenovector-based vaccine dose [85]. This diverging observation may be explained by the exclusive consideration of patients with solid cancers who did not receive immunotherapy and the resulting similarity to the healthy population [85]. Referring to additional data from immunocompetent individuals, a similarly broad CD4+ and CD8+ T cell response became evident after a third dose of a mRNA vaccine, compared to the second dose [23]. Furthermore, as discussed above, a third shot induces CD8+ T cell responses against conserved epitopes within the spike protein, including VOC such as Omicron [23]. Similar results in cancer patients would certainly underscore the beneficial effects of booster vaccinations in this vulnerable group. However, at present, no comparable data are available, and further trials, ideally including in-depth analyses of virus-specific T cells, are urgently required.

Additional experiments focusing on adaptive immune responses after SARS-CoV-2 booster vaccination were performed in patients with hematologic cancers, such as multiple myeloma, lymphocytic leukemia, and B cell non-Hodgkin’s lymphoma. These groups are known to develop a weaker humoral immunity and similar T cell responses, compared to patients with solid cancers. Importantly, booster vaccination with a mRNA vaccine results in significant increases in humoral and cellular immune responses, including VOC [86,87]. However, compared to healthy individuals, the SARS-CoV-2-specific adaptive immunity may still be impaired, resulting in a higher susceptibility towards breakthrough infections. In this regard, and according to national vaccination strategies, a fourth vaccination against SARS-CoV-2 is recommended for endangered patients in many countries. Indeed, the application of a fourth mRNA vaccine dose induces not only increased titers of neutralizing antibodies, but also higher levels SARS-CoV-2-specific CD4+ and CD8+ T cell responses against the currently predominant VOC Omicron in patients with hematological malignancies [88]. Moreover, an interesting case report by Atanackovic et al. investigated the influence of multiple mRNA vaccine doses in a patient with B cell lymphoma suffering from significantly impaired antibody responses after vaccination due to a chemotherapy-related B cell depletion [89]. Antibodies against SARS-CoV-2 occurred only after the off-label administration of a fifth and sixth mRNA vaccine dose post-B cell recovery, while in vitro peptide stimulation revealed strong virus-specific CD4+ and CD8+ T cell responses with the ability to cross-recognize Omicron [89]. Yet, despite this promising observation, a fifth and sixth dose is currently reserved for off-label use and has to be evaluated in larger cohorts to confirm the described effects. Nonetheless, the need for booster vaccination against SARS-CoV-2 in cancer patients is congruent with the observations in influenza vaccination, where weak immune responses elicited by a single dose can be improved with the help of repeated vaccinations [90].

#### 3.2.3. Discordance between the Vaccine-Induced SARS-CoV-2-Specific Humoral and Cellular Immune Response

Another important aspect in patients with cancer is the discordance between humoral and cellular immune responses. Indeed, individuals without vaccine-induced seroconversion may develop a SARS-CoV-2-specific cellular immunity after vaccination, offering protection against severe COVID-19 [91]. For example, in patients with multiple myeloma, seroconversion rates after two mRNA-based SARS-CoV-2 vaccine doses are significantly reduced, compared to healthy subjects, particularly in the case of anti-CD38 therapy [92,93]. Regarding virus-specific T cells, only 35% of seronegative patients versus 96% of seropositive patients showed virus-specific CD4+ T cell responses, whereas CD8+ T cell responses were on a quite similar level in both cohorts (Figure 2C) [94]. Nevertheless, detectable SARS-CoV-2-specific CD8+ T cells in the seronegative patients were largely not polyfunctional with CD8+ T cells that produce predominantly only IFN-γ [94]. Extending these results, a recent study by Atanackovic et al. revealed the development of virus-specific CD4+ and CD8+ T cells in B cell lymphomas after treatment with chimeric antigen receptor T (CAR-T) cells and a third SARS-CoV-2 mRNA vaccine dose, while antibody responses are lacking [95]. Similarly, patients undergoing anti-CD20 treatment generate vaccine-induced T cells, even if antibodies are undetectable [78,96]. Overall, the discordance between humoral and cellular immune responses to SARS-CoV-2 vaccination may warrant routine serologic testing of patients with hematologic malignancies. This strategy could support the identification of individuals with weak antibody responses who might particularly benefit from early booster vaccination to augment the SARS-CoV-2-specific cellular immunity.

#### 3.2.4. Influence of Cancer Treatment on the Vaccine-Induced Cellular Immune Response

Cancer treatment, such as steroid therapy, checkpoint-inhibitor therapy, anti-CD20 treatment or hematopoietic cell transplantation (HCT), is able to result in the suppression of both cellular and humoral immune responses following vaccination (Figure 2D) [97]. For instance, recent data for vaccines against influenza and varicella-zoster viruses suggest that cancer patients under anti-CD20 treatment do not develop sufficient humoral immune responses [98], while virus-specific T cell responses are present [99]. This is consistent with already introduced results in multiple sclerosis patients treated with anti-CD20 antibodies, revealing detectable virus-specific CD4+ and CD8+ T cells after SARS-CoV-2 mRNA vaccination [96]. Next, cancer patients receiving HCT are known to develop a lower immune response to vaccines in the first years after transplantation, including vaccines against typical pathogens of respiratory infections, such as influenza viruses or streptococcus pneumoniae [100]. Similarly, for SARS-CoV-2, mRNA vaccination of HCT recipients resulted in a humoral and/or cellular immune response in only 75% of the tested patients [101]. These findings are complemented by analyses from Lindemann et al. [102]. Here, in line with previous studies [103,104], HCT recipients displayed a significantly reduced humoral and cellular immunity against SARS-CoV-2, following two doses of mRNA vaccination (Figure 2D) [102]. In contrast, Harrington et al. showed that both CD4+ and CD8+ T cell responses are enhanced by a second vaccine dose with higher expression levels of IFN-γ and TNF [105]. Consequently, while pointing towards the need for early and repeated booster vaccinations (Figure 2E), further studies in larger cohorts are necessary to validate these observations and clarify the impact of additional vaccine administrations.

#### 3.2.5. Breakthrough Infections in Vaccinated Cancer Patients

Due to circulating VOC, such as the currently predominant omicron variant, breakthrough infections have been reported to occur in both immunocompetent and immunocompromised individuals [106,107]. In particular, the VOC Omicron has multiple amino acid mutations within the spike protein, which promote the ability to escape the antibody response induced by vaccination or infection [25,26,27]. Regarding the T cell-mediated immunity, SARS-CoV-2-specific CD4+ and CD8+ T cells induced by both vaccination and infection in healthy individuals have been shown to be cross-reactive against VOC, including Omicron [23,56,108,109]. Moreover, disease severity appears to be mild to moderate in individuals with virus-specific cellular responses, highlighting the protection against severe COVID-19 by strong and polyfunctional T cells [107]. However, Naranbhai et al. described a reduced T cell immunity towards Omicron after vaccination or infection in selected individuals, especially on the CD8+ T cell level, that might be explained by an escape from HLA binding [109]. This is an important observation, specifically in the context of frequently impaired humoral immune responses in cancer patients, since these results might also apply to this patient group and, therefore, lead to a significantly increased susceptibility towards VOC. Unfortunately, to date, no comparable studies are available for cancer patients. Nonetheless, considering these potential risks, the development of second-generation vaccines that induce potent T cell responses targeting both spike and non-spike proteins of SARS-CoV-2 could be an effective tool for protection against current and emerging viral variants.

## 4. SARS-CoV-2-Specific T Cells in People Living with HIV (PLWH)

### 4.1. Cellular Immunity during and after SARS-CoV-2 Infection

**Figure 3:**
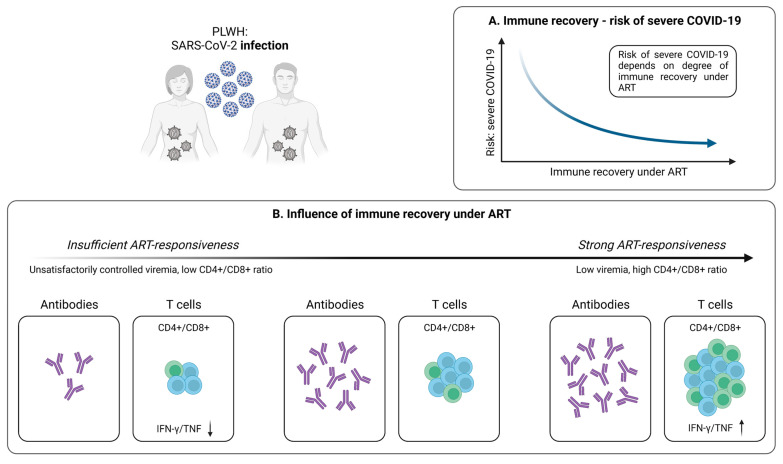
Infection-induced SARS-CoV-2-specific adaptive immune responses in PLWH. (**A**) Relationship between immune recovery under antiretroviral therapy (ART) and the risk of severe COVID-19 in PLWH. (**B**) Depiction of the influence of immune reconstitution under ART on the generation of virus-specific humoral and cellular immune responses upon SARS-CoV-2 infection in PLWH. Created with BioRender.com, 2 February 2023.

HIV is characterized by a cellular immune deficiency with progressive CD4+ T cell loss, resulting in an increased susceptibility towards various infections [110]. This also appears to be the case for SARS-CoV-2, with PLWH being at higher risk for severe courses of COVID-19 accompanied by hospitalization [111]. Importantly, these events appear to predominantly affect PLWH with low CD4+ T cell counts and insufficient viral suppression (Figure 3A) [111,112,113]. 

#### 4.1.1. SARS-CoV-2-Specific T Cells in PLWH

Although PLWH represent a particularly vulnerable patient population, the impact of T cell-mediated immunity in case of SARS-CoV-2 infection has not been fully elucidated in this cohort. Early results indicate impaired frequencies of SARS-CoV-2-specific CD4+ T cells producing IFN-γ and TNF-α in patients with unsuppressed HIV infection, compared to PLWH with controlled viremia (Figure 3B) [30]. However, no significant differences on the CD8+ T cell level were identified [30]. Moreover, Adachi et al. observed a transient decrease in CD4+ T cell, CD8+ T cell, and total lymphocyte counts during SARS-CoV-2 infection [114]. Nevertheless, this observation is not specific for HIV/SARS-CoV-2 coinfection, but more likely a phenomenon that also occurs in individuals without HIV, pointing towards an association between decreased lymphocyte subsets and a more severe course of COVID-19 [115]. Interestingly, the CD4+/CD8+ ratio did not change in PLWH during SARS-CoV-2 infection, compared to healthy individuals who are characterized by an increase in this parameter [114,115]. Given that a low CD4+/CD8+ ratio is predictive of adverse clinical outcomes in PLWH, it could serve as a potential tool for evaluating the course of COVID-19 in these patients, thus providing an additional option in risk stratification [116,117].

Despite these findings, a detailed understanding of the SARS-CoV-2-specific T cell-mediated immunity in PLWH is still missing, especially in those with unsuppressed viremia. However, as some commonalities between PLWH and healthy individuals are clearly detectable, these patients could particularly benefit from vaccination against SARS-CoV-2 to induce a strong virus-specific cellular immune response.

#### 4.1.2. Antiretroviral Therapy Is Associated with Stronger SARS-CoV-2-Specific T Cell Responses

Although antiretroviral therapy (ART) does not allow for the eradication of HIV, it enables a significant reduction of the morbidity and mortality resulting from HIV infection [118]. This also applies to patients co-infected with HIV and SARS-CoV-2 receiving ART, where suppressed HIV is associated with reduced COVID-19 severity and mortality (Figure 3A) [119]. Importantly, untreated PLWH exhibits a less effective immune response to SARS-CoV-2, compared to PLWH under ART and low viremia (Figure 3B) [30,119,120]. Indeed, it has recently been shown that, in contrast to uncontrolled HIV infection, PLWH with suppressed viral load exhibit similar SARS-CoV-2-specific CD4+ and CD8+ T cell responses with analogous cytokine expression, compared to healthy individuals [30]. In line, Alrubayyi et al. were able to detect the development of SARS-CoV-2-specific CD4+ and CD8+ T cells in PLWH on ART and further describe a correlation between the CD4+/CD8+ ratio and the magnitude of SARS-CoV-2-specific T cell responses [120]. Of note, the development of virus-specific antibodies in PLWH also seems to be influenced by the frequency of CD4+ T cells (Figure 3B) [121]. Collectively, it therefore appears that successful ART substantially improves the adaptive immune response in these patients. Moreover, especially the CD4+/CD8+ ratio may be used as an important marker for assessing the individual risk of severe COVID-19 in PLWH and to determine a suitable vaccination strategy for individuals with inadequate responses to antiviral therapy.

### 4.2. Cellular Immunity Following SARS-CoV-2 Vaccination

In line with these results from SARS-CoV-2 infection, PLWH with low CD4+ T cell counts are at risk of being hyporesponsive to vaccination and having less durable immune responses to various vaccines [122,123]. In the context of the current COVID-19 pandemic, the immunogenicity of vaccination against SARS-CoV-2 in PLWH, intended to provide protection against severe disease, has not been fully elucidated. However, emerging data provide important insights into this issue.

#### 4.2.1. Robust Vaccine-Induced T Cell Immunity in PLWH

**Figure 4:**
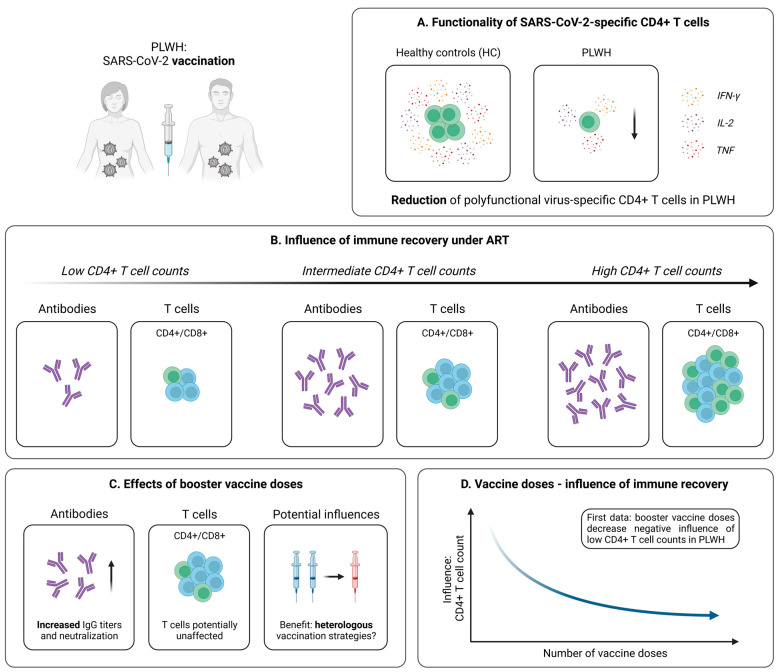
Vaccine-induced SARS-CoV-2-specific adaptive immune responses in PLWH. (**A**) Differences in the generation of SARS-CoV-2-specific polyfunctional (IFN-γ+IL-2+TNF+) CD4+ T cells following vaccination in PLWH and healthy controls (HC). (**B**) Depiction of the influence of immune reconstitution under ART on virus-specific humoral and cellular immune responses induced by SARS-CoV-2 vaccination in PLWH. (**C**) Collection of early data addressing the effect of booster vaccine doses in PLWH. (**D**) Illustration of first results pointing towards an inverse relationship between the influence of immune recovery under antiretroviral therapy (ART) and the number of vaccine doses in PLWH. Created with BioRender.com, 2 February 2023.

Within the last two years, several studies investigated the development of antibody responses after SARS-CoV-2 mRNA or vector vaccination in PLWH and revealed kinetics similar to those in healthy individuals, especially under effective antiviral treatment [124,125,126,127]. However, at present, knowledge of vaccine-induced T cell responses remains limited. Woldemeskel et al. performed early analyses within a small cohort of PLWH and healthy controls after two mRNA vaccine doses [128]. Via the application of an IFN-γ ELISPOT, the authors detected SARS-CoV-2-specific T cell responses with comparable magnitudes in both groups, which were directed against similar sections within the spike protein. More detailed experiments from Gao et al. revealed a quite similar picture; however, slight impairments especially concerning the T cell (poly-) functionality became evident (Figure 4A) [129]. Of note, analogous results could be obtained for immunization with the vector-based vaccine AZD1222 [130]. Finally, T cell responses seem to be preserved up to six months after the second vaccine dose, albeit a slight decrease over time becomes evident in PLWH irrespective of the underlying vaccine platform [129,131,132].

#### 4.2.2. Influence of Immune Recovery under ART on the Vaccination Outcome

In light of these surprisingly positive results, it is important to note that the studies mentioned above predominantly included PLWH under effective ART displaying more or less inconspicuous median CD4+ T cell counts. For this reason, it remained unclear whether PLWH without such a strong immune reconstitution are able to generate similar robust SARS-CoV-2 vaccine-induced cellular immune responses. Several studies addressed this question by dividing their patient cohorts into subgroups based on the individual CD4+ T cell count. Interestingly, an investigation of Antinori et al. detected IFN-γ and IL-2 production upon stimulation with spike peptides in the blood of PLWH characterized by a low (<200 cells/µL), medium (200–500 cells/µL), and high (>500 cells/µL) CD4+ T cell count [133]. However, patients with a CD4+ T cell count < 200 cells/µL displayed significantly reduced levels of these cytokines at all post-vaccination time points, suggesting impaired mRNA vaccine-elicited T cell responses in this cohort [133]. In contrast, the results obtained in patients with CD4+ T cell values > 500 cells/µL were almost identical to those derived from healthy individuals [133]. Accordingly, after adjustment for major confounders (e.g., age, CD4+ nadir, or years since HIV diagnosis), a low CD4+ T cell count was linked to significantly impaired humoral and cellular immune responses after SARS-CoV-2 mRNA vaccination (Figure 4B) [133]. Further support for these results is delivered by additional studies with similar approaches considering the effect of a low CD4+ T cell count on the vaccination success in PLWH [134,135].

#### 4.2.3. Optimization of SARS-CoV-2 Immunization Strategies in PLWH

Due to these findings, an adjusted immunization strategy for PLWH with unsuppressed viremia seems to be necessary. Several tools, including vaccination with specific formulations or booster vaccine doses, have already been established in the context of other vaccinations [136,137,138]. Regarding SARS-CoV-2, evidence for a beneficial effect of additional vaccine shots in PLWH is provided by Vergori et al. [139]. Here, the authors assessed adaptive immune responses following a third dose of mRNA vaccination in a large cohort of PLWH and healthy individuals. While the humoral response was on a quite comparable level in both groups, PLWH displayed a significantly reduced T cell-related production of IFN-γ upon stimulation with spike-specific peptides [139]. Of note, when comparing subgroups of PLWH based on their CD4+ T cell recovery during ART, as explained above, a trend towards attenuated vaccine-induced immune responses in patients with low CD4+ T cell values became evident [139]. However, after adjustment for relevant confounders, no significant association between the count of CD4+ T cells and SARS-CoV-2-specific antibody titers, neutralization, or T cell responses could be identified [139]. Furthermore, it seemed that PLWH receiving a heterologous vaccination with two shots of BNT162b2, followed by mRNA-1273 as a third dose, generated superior humoral responses, compared to those receiving three identical vaccine doses (Figure 4C) [139]. In contrast, no clear effect of mixed vaccine formulations was observed for the SARS-CoV-2-specific cellular immunity [139]. Predominantly similar observations are reported from trials analyzing the effect of heterologous vaccination in healthy individuals [140,141,142]. Taken together, these findings indicate that a third vaccine dose is able to induce a solid immune response in PLWH, while notably diminishing the negative impact of low CD4+ T cell counts observed after the second vaccine dose (Figure 4D).

## 5. SARS-CoV-2-Specific T Cells in Solid Organ Transplant Recipients (SOT)

### 5.1. Cellular Immunity during and after SARS-CoV-2 Infection

Driven by the rapid and global spread of COVID-19, the increased risk of infection associated with immunosuppressive therapies in solid organ transplant recipients (SOT) has moved to the center of medical and public interest. Nevertheless, the virus-specific T cell immunity in SOT and its interaction with other domains of the adaptive immune system in SARS-CoV-2 infection remains incompletely characterized.

#### 5.1.1. SARS-CoV-2-Specific T Cells in SOT

**Figure 5:**
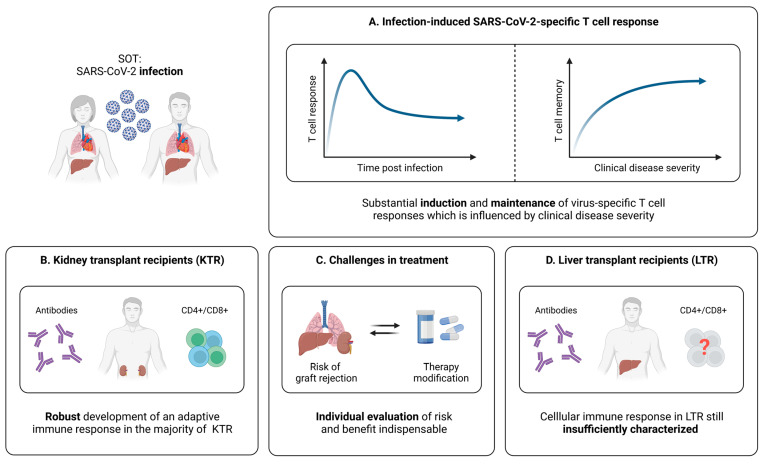
Infection-induced SARS-CoV-2-specific adaptive immune responses in SOT. (**A**) Characteristics of the SARS-CoV-2-specific cellular immune response in infected and convalescent SOT. (**B**) Brief summary of current knowledge about adaptive immune responses elicited by SARS-CoV-2 infection in kidney transplant recipients (KTR). (**C**) Illustration of challenges in the clinical management of SOT infected with SARS-CoV-2. (**D**) Brief summary of current knowledge about adaptive immune responses elicited by SARS-CoV-2 infection in liver transplant recipients (LTR). Created with BioRender.com, 2 February 2023.

An early important study focusing on the development of a cellular immunity after SARS-CoV-2 infection in SOT (kidney, heart, liver) was performed by Fava et al. [143]. Via the application of a multicolor FluoroSpot assay, the authors detected slightly lower frequencies of cytokine producing virus-specific T cells in a cohort of 28 SOT, compared to 16 immunocompetent controls at early stages after infection. In addition, SARS-CoV-2-specific T cell responses in SOT seemed to be mainly focused on the viral spike protein [143]. Importantly, about one month after symptom onset, the overall proportions of SARS-CoV-2-specific T cells producing, e.g., IFN-γ, upon stimulation were on a quite similar level in SOT and the controls (Figure 5A) [143]. Thieme et al. complemented these findings by revealing similar frequencies of both CD4+ and CD8+ T cells activated upon stimulation with several viral peptides in SOT (kidney, pancreas, lung) and controls [144]. Moreover, SOT exhibited a comparable T cell functionality, as well as congruent frequencies of SARS-CoV-2-specific CD4+ and CD8+ T cells with a memory phenotype [143]. However, due to the limited sample size, these observations need to be evaluated carefully and should be validated in larger cohorts.

#### 5.1.2. Relation between SARS-CoV-2-Specific T Cell Responses and the Clinical Course of Infection in SOT

As already discussed, strong CD4+ and CD8+ T cell responses are associated with an efficient antiviral defense in healthy individuals [41,44,45]. For SOT, the first analyses in this regard were performed by Del Bello et al., revealing that the overall count of CD3+ and CD8+ T cells is significantly reduced in SOT (kidney, liver, heart, pancreas) with severe COVID-19 [145]. In contrast, these patients displayed a higher proportion of activated CD4+ T cells, which seemed to be accompanied by significantly elevated frequencies of regulatory T cells (T_regs_) [145]. Overall, these results suggest differences between mild and severe courses of SARS-CoV-2 infection in SOT at the T cell level, which resemble previous reports from healthy individuals [145,146,147,148]. Nonetheless, it is important to note that a major limitation of this study consists of the global assessment of T cells, without taking into account whether these cells are SARS-CoV-2-specific or not. Addressing this problem, a more comprehensive approach was applied by Fava et al., who compared the adaptive memory formation six months after SARS-CoV-2 infection in SOT (kidney, heart, liver, lung) and immunocompetent controls with different disease severities [149]. Here, the overall T cell response was quite similar in SOT and controls, while statistically significant differences became evident mainly depending on the course of disease (Figure 5A) [149]. The frequencies of IFN-γ+, IL-2+, or IFN-γ+IL-2+ T cells were significantly lower for both SOT and controls with mild COVID-19, compared to severe disease, whereas more ambiguous results were obtained for IL-21 producing cells [149]. Of note, several subtle impairments of the immune response in SOT could be identified, among them, faster decreasing frequencies of virus-specific T cells, when comparing the levels six months post-infection to those at earlier time points [149]. However, combined with analyses concerning the antibody and B cell response, the results from this investigation suggest that SOT are capable of generating a long-lasting immunity after SARS-CoV-2 infection, which is mainly influenced by the disease severity (Figure 5A).

#### 5.1.3. SARS-CoV-2-Specific T Cell Immunity in Kidney Transplant Recipients (KTR)

Kidney transplant recipients (KTR) represent a major proportion of SOT. Hence, studies currently focusing on isolated subgroups of SOT mostly investigate adaptive immune responses during and after SARS-CoV-2 infection in these patients. In a first small trial, it could be demonstrated that KTR recovered from COVID-19 display robust T cell responses after stimulation with different viral peptides [150]. More detailed analyses from Bertrand et al. within a larger cohort confirmed these results and pointed towards an efficient development of a cellular immunity in KTR upon SARS-CoV-2 infection [151]. Of note, a longitudinal observation revealed the persistence of virus-specific T cell responses up to ten months post-infection, while antibody levels were barely detectable at that time [151].

Important additional experiments have been performed by Charmetant et al. with the aim to compare adaptive immune responses in KTR elicited by SARS-CoV-2 infection and vaccination [152]. As expected and consistent with the findings in healthy individuals, CD4+ and CD8+ T cells derived from vaccinated KTR were only directed against spike, while convalescent individuals displayed additional responses towards other viral proteins [23,152]. However, the total number of virus-specific CD4+ and CD8+ T cells was very similar in both groups [152]. In terms of T cell functionality, SARS-CoV-2-specific CD4+ T cells derived from convalescent KTR displayed a significantly elevated IFN-γ production upon in vitro stimulation [152]. In contrast, this difference could only be detected as a trend for CD8+ T cells [152].

Taken together, it seems that KTR are able to mount overall robust SARS-CoV-2-specific humoral and T cell responses upon natural infection (Figure 5B). Nonetheless, it is important to note that the management of immunosuppressive therapies during the infection period varies highly between the described studies. It is, therefore, difficult to determine whether single modulations can lead to particularly prominent improvements or impairments of the SARS-CoV-2-specific cellular immunity. Until further data are available, decisions regarding a potential reduction or cessation of the immunosuppressive treatment, in the case of SARS-CoV-2 infection, should therefore be carefully based on the individual patient’s condition and risk of graft rejection (Figure 5C).

#### 5.1.4. SARS-CoV-2-Specific T Cell Immunity in Liver Transplant Recipients (LTR)

Another important group within SOT are liver transplant recipients (LTR). To date, most studies on the adaptive immunity in this cohort are limited to the humoral response upon SARS-CoV-2 infection. Recent investigations point towards the generation and maintenance of antibodies with moderate impairments, especially concerning IgG directed against the nucleocapsid protein [153,154,155]. Yet, knowledge of the virus-specific cellular immunity during and after SARS-CoV-2 infection in this patient population is very limited. At present, only one study assessed T cell responses in a larger cohort of convalescent LTR [156]. Here, Fernandez-Ruiz et al. included 31 LTR in a range of 30 to more than 180 days post-infection, as well as 30 age- and time point-matched immunocompetent controls. While the vast majority of LTR displayed SARS-CoV-2-specific T cell responses, the frequencies of virus-specific CD8+ T cells seemed slightly lower, compared to CD4+ T cells [156]. Of note, no differences could be detected between LTR and healthy individuals, regarding the strength of T cell responses against the spike, nucleocapsid, and membrane protein of SARS-CoV-2 [156]. In sum, SARS-CoV-2 infection seemed to efficiently induce a cellular immune response in LTR, which was also detectable at later time points during convalescence [156]. However, further studies confirming these results and performing in-depth analyses of virus-specific T cells are necessary to broaden our knowledge about the cellular immunity elicited by SARS-CoV-2 infection and potential influences of immunosuppressive medications within the group of LTR (Figure 5D).

### 5.2. Cellular Immunity Following SARS-CoV-2 Vaccination

Due to the increased risk of infection associated with immunosuppressive therapies, SOT could particularly benefit from a vaccine-induced boost of the adaptive immune response. However, in contrast to healthy individuals, the efficacy of SARS-CoV-2 vaccination is far less well-investigated in this patient group.

#### 5.2.1. Vaccine-Induced SARS-CoV-2-Specific T Cell Immunity in SOT

**Figure 6:**
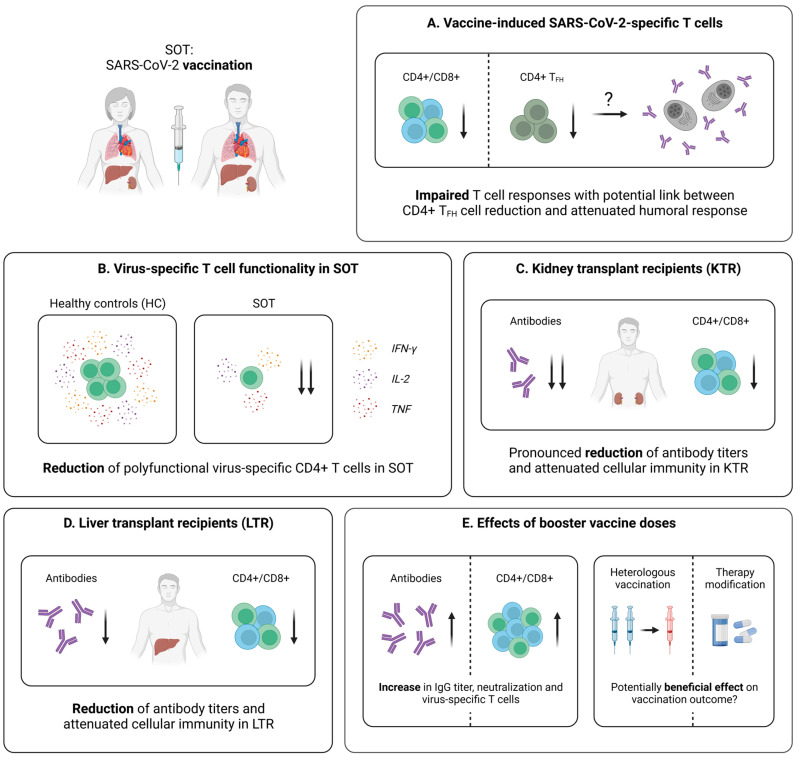
Vaccine-induced SARS-CoV-2-specific adaptive immune responses in SOT. (**A**) Characteristics of the vaccine-induced cellular immunity in SOT. (**B**) Comparison of the amounts of polyfunctional virus-specific CD4+ T cells in vaccinated SOT and controls. (**C**) Brief summary of current knowledge about adaptive immune responses elicited by SARS-CoV-2 vaccination in kidney transplant recipients (KTR). (**D**) Brief summary of current knowledge about adaptive immune responses elicited by SARS-CoV-2 vaccination in liver transplant recipients (LTR). (**E**) Collection of early data addressing the effect of booster vaccine doses in SOT. Created with BioRender.com, 2 February 2023.

Several studies addressing the cellular immunity following SARS-CoV-2 mRNA vaccination demonstrated lower response rates in SOT (kidney, liver, lung, heart), compared to healthy individuals [157,158]. In addition, Schmidt et al. point towards a higher effectiveness of heterologous vaccine regimens (combination of AZD1222 and mRNA vaccine), when it comes to the induction of for instance CD4+ T cells in SOT (kidney, heart, lung, liver) [159]. A more detailed understanding of the impact of immunodeficiency on the profile of vaccine-induced SARS-CoV-2-specific T cells is provided by a study of Gao et al. [129]. Here, SOT (liver, kidney, pancreas) and other immunocompromised cohorts were analyzed after a first and second mRNA vaccine dose. Importantly, SOT displayed the lowest overall T cell response assessed by an IFN-γ ELISPOT assay, compared not only to healthy controls, but also for example to patients with primary immunodeficiencies or HIV infection [129]. When divided into CD4+ and CD8+ T cell responses, the SOT group was characterized by reduced frequencies of both populations five months after the second vaccine dose [129]. Furthermore, SOT showed the lowest frequency of cross-reactive (pre-existing) CD4+ T cells, which were proven to be an important factor linked to efficient spike-specific CD4+ T cell responses early after vaccination [129]. Combined with similar negative results in functional assays, these data suggest a significantly attenuated and non-coordinated cellular response following two mRNA vaccine doses in SOT (Figure 6A,B) [129]. Studies from Ferreira et al. [160] complemented these findings by comparing the T cell responses in SOT (kidney, liver, lung, pancreas, heart) after infection and mRNA vaccination. Here, SARS-CoV-2 infection seemed to generate a stronger T cell immunity in SOT, assessed via the quantification of polyfunctional spike-specific CD4+ T cells [160]. However, the vaccinated cohort in this study was characterized by significantly older age, which may explain the weaker cellular immune response, as previously described for other vaccinations in the elderly [161,162].

Taken together, the current results point towards an impaired T cell response in SOT following SARS-CoV-2 vaccination, with a potentially less robust development, compared to natural infection. Addressing this problem, several reports propose different strategies with the aim of enhancing the vaccine-elicited immunity in these patients, among them, booster vaccine doses and temporary therapy adjustments [152,159,163,164,165,166]. Promising examples of these potential solutions applied in the groups of KTR and LTR will be further discussed in the following sections.

#### 5.2.2. Vaccine-Induced SARS-CoV-2-Specific T Cell Immunity in KTR

In KTR, an impaired T cell immunity following SARS-CoV-2 mRNA vaccination has been observed, when compared to healthy individuals (Figure 6C) [167,168]. As revealed by Sattler et al., the overall frequency of individuals developing spike-specific CD4+ T cells after the second mRNA vaccine dose were comparable in an age-matched cohort of KTR and healthy controls, while the magnitude of these T cell responses was significantly reduced in KTR [169]. In addition, the proportion of individuals with detectable virus-specific CD8+ T cells was strikingly lower within the patient cohort, and spike-specific T cells derived from KTR displayed a reduced effector cytokine production (e.g., IFN-γ or TNF) [169]. Furthermore, KTR were characterized by significantly decreased levels of polyfunctional (IFN-γ+TNF+IL-2+) spike-specific T cells, compared to healthy controls [169]. Finally, these results were complemented by RNA sequencing, pointing towards a general decrease of several features linked to cellular activation (e.g., genes associated with signaling, inflammation and metabolism) in KTR [169].

Next, important investigations on the differences in the virus-specific B and T cell immunity between KTR with and without neutralizing antibody responses, following a second mRNA vaccine dose, revealed that T_FH_ cells were significantly reduced in patients without seroconversion, correlating with neutralizing antibodies and RBD-specific B cells (Figure 6A) [152]. These findings provide a potential mechanism determining the development of a robust serological response upon vaccination and, therefore, emphasize the importance of a functional T cell immunity. Interestingly, the KTR with low T_FH_ frequencies included in this study received significantly higher doses of mycophenolate mofetil (MMF), compared to those with higher frequencies of T_FH_ cells [152]. This is consistent with the observations made in SOT, describing MMF as a factor that negatively influences immune responses after mRNA- or adenovector-based SARS-CoV-2 vaccination [129,170,171,172]. The strong interference can be explained by the pharmacodynamics of MMF: via the selective inhibition of the inosine monophosphate dehydrogenase (IMPDH), the drug represses the proliferation of T and B lymphocytes, which are highly dependent on the de novo synthesis of purine nucleotides [173,174]. Accordingly, the administration of a fourth mRNA vaccine dose in seronegative KTR with an intermittent mycophenolate hold favors the expression of proliferation and activation markers, such as Ki-67 and PD-1, on SARS-CoV-2-specific T cells, while cytokine production and memory differentiation, as well as the frequencies of spike-reactive CD4+ T cells, appear to remain stable [164].

This observation may have clinical implications for patients receiving immunosuppressive therapies: in addition to the application of booster vaccine doses, a temporary modification or hold of the treatment could improve the vaccine-elicited adaptive immune response in this endangered population. However, further studies are required to investigate this effect with other immunosuppressants and to determine whether a reduction in medication is sufficient for achieving the same effect as complete cessation. At present, especially due to the potential association with an increased risk of graft rejection, this strategy should be undertaken only under rigorous risk–benefit evaluation for each individual patient.

#### 5.2.3. Vaccine-Induced SARS-CoV-2-Specific T Cell Immunity in LTR

Similar as for KTR, reports on the vaccine-induced adaptive immunity in LTR mostly focus on the humoral immune response [170,171,175,176,177], while only few studies investigate the SARS-CoV-2-specific T cell immunity (Figure 6D). Of note, emerging data clearly indicate that the cellular response is impaired after a second vaccine dose, but can be enhanced with the help of further booster vaccinations [165,166,178,179]. An important early work by Ruether et al. assessed spike-specific T cell responses in LTR, following a second vaccine dose (BNT162b2, mRNA-1273, or AZD1222), with the help of a commercial IFN-γ release assay (IGRA) [178]. In fact, the response rates, as well as the median IFN-γ concentration, were significantly reduced in LTR, compared to the control cohort [178]. Additionally, a high discordance between the humoral and cellular response became evident in LTR [178]. This is consistent with findings by studies from Harberts et al. [165] and Davidov et al. [166], demonstrating an improved, but still compromised, spike-specific T cell response, following a third or fourth mRNA vaccine dose in LTR (Figure 6E). Nonetheless, further studies with extended methods are urgently required to broaden our understanding of vaccine-elicited T cell responses in this patient group.

## 6. Conclusions

Taken together, knowledge of the SARS-CoV-2-specific cellular immunity in immunocompromised patients evolves quickly, but is still limited. Nevertheless, when comparing the groups of cancer patients, PLWH, and SOT discussed in this review, several major commonalities become evident.

First, individual conditions and therapies appear to strongly influence the development of virus-specific T cell responses in all cohorts. While this mainly concerns the different disease subtypes in cancer patients (e.g., solid cancers and hematological malignancies), the response in PLWH particularly depends on the degree of immune reconstitution under ART. On the other hand, the generation and maintenance of SARS-CoV-2-specific T cells in SOT is highly variable and linked to the handling of the individual immunosuppressive therapy. Consequently, further studies determining guidelines for the optimal management of these factors are urgently needed, concerning, for instance, specific therapy adjustments or adapted vaccination schemes. Second, the analyzed patient groups often display a certain discordance between humoral and cellular immune responses. These findings underscore the importance of comprehensive approaches exceeding the isolated consideration of antibody responses as a correlate of vaccination success and protection. In line, T cell assays could be a valuable tool in the evaluation of individual patients with an uncertain immune status upon infection or vaccination. For this reason, the development and improvement of approaches suitable for clinical implementation, among them, interferon-γ release assays (IGRA), could lead to significant diagnostic and preventive benefits, which would certainly exceed the field of SARS-CoV-2. Third, the current data imply booster vaccinations as important measures leading to an enhanced SARS-CoV-2-specific immunity in immunocompromised patients. While first results suggest a particularly improved humoral response, a positive effect on virus-specific T cells is likely, but less well-characterized and, therefore, requiring further analyses. Nonetheless, also referring to next-generation vaccines, which elicit an improved immunity against the Omicron variants of SARS-CoV-2, additional booster doses should be considered in the case of insufficient immune responses. Fourth, important aspects, such as the phenotype, functionality or epitope repertoire of T cells induced by SARS-CoV-2 infection and vaccination in immunocompromised patients remain largely unexplored. Given their significance for the evaluation of the long-term immune memory, the risk of breakthrough infections, or the protection against existing and emerging VOC, more detailed insights into these topics are of great scientific and clinical interest.

Collectively, the here discussed results might assist physicians in the guidance of immunosuppressed patients, concerning the management of infection or the benefit of (booster) vaccinations. Moreover, since the overall attenuated immunity is a general phenomenon in this complex population, a variety of the novel findings described for SARS-CoV-2 could, at least partially, also apply to other infectious diseases and vaccinations. Finally, the significant number of unanswered questions identified in the previous chapters emphasizes the necessity of additional investigations to complement the current findings and to support a comprehensive understanding of the immunological particularities and challenges in these vulnerable patients.

## Data Availability

Not applicable.
